# Extending the COSS Model to Youth Mental Health: Outcomes and Learnings from the Albury Project Mental Health Pilot

**DOI:** 10.3390/ijerph22121780

**Published:** 2025-11-25

**Authors:** Tammy Hand, David MacKenzie, Peter Gill, Jascha Zimmermann, Kate McGrath, Deagan Jackson

**Affiliations:** 1 Upstream Australia Ltd., North Melbourne 3051, Australia; david.mackenzie@upstreamaustralia.org.au; 2 College of Sport, Health and Engineering, University of Victoria, Melbourne 3000, Australia; peter.gill@vu.edu.au; 3 Department of Biomedical, Health and Exercise Sciences, Swinburne University, Melbourne 3000, Australia; jzimmermann@swinburne.edu.au; 4 Yes Unlimited, Albury 2640, Australia; kate.mcgrath@yesunlimited.com.au (K.M.);

**Keywords:** youth mental health, early intervention, COSS Model, population screening, psychological distress, community collaboration, collective impact, Australia, adolescent wellbeing, Kessler Psychological Distress Scale

## Abstract

This article presents key findings and learnings from the Albury Project Mental Health Pilot, a six-month exploratory opportunity designed to extend the Community of Schools and Services (COSS) Model to proactively identify and support young people experiencing psychological distress in a regional Australian community. Working within the established COSS Model architecture and using population-level screening via the Australian Index of Adolescent Development (AIAD) survey, the pilot focused on a previously unreached and unsupported cohort of young people with high or very high scores on the Kessler Psychological Distress Scale (K10) who were not engaged with existing mental health services. The support involved brief, tiered interventions tailored to individual needs. Short-term outcomes demonstrated improvements in K10 scores for the majority of participants, with many moving out of the K10 at-risk range. Medium-term data further showed sustained improvements in psychological distress and personal wellbeing for a substantial proportion of the cohort. The pilot identified a ‘hidden cohort’ and demonstrated that low-resource, brief interventions are capable of achieving high-impact outcomes within the existing COSS Model collective impact framework. While short-term, these findings suggest the COSS Model’s adaptability and promise as a cost-efficient early intervention platform for youth mental health in broader community settings.

## 1. Introduction

Youth mental health is considered a public health concern globally, with a significant proportion of mental health challenges emerging during adolescence. According to a recent national survey in Australia, nearly 40 percent of people aged 16–24 years reported experiencing a mental health disorder in the past month [1]. The rate of mental health disorders in 4–17-year-olds was 14 percent, most commonly attention deficit/hyperactivity disorders and anxiety disorders [2]. For young Australians aged 5–17 years, suicide is the leading cause of death. Youth mental health services are currently fragmented across different settings and funding models, and across different age groups and health conditions [3]. This lack of integration leads to inefficiencies and poorer outcomes.

Australia’s national youth mental health foundation, headspace, established in 2006, provides early intervention and primary mental health services for young people aged 12–25 years. Services are delivered through a network of more than 100 centres nationwide, complemented by online and telephone-based counselling platforms [4,5]. The Australian Government has been the primary funder, progressively increasing investment in the service network—from $32 million in 2018 to $49.9 million in 2021 and $59.9 million in 2022 [4,5]. Since its inception, *headspace* expanded from 10 centres in 2007 to over 100 within a decade, directly assisting approximately 88,500 young people in 2017–18, with an additional 33,700 supported via online and telephone services [4]. The majority of clients in that year were adolescents (59% aged 12–17 years), with a further 21% aged 18–20 years and 18% aged 21–25 years [4]. Despite the rapid expansion of *headspace* centres, significant service pressures remain, with reports of long waiting lists—particularly exacerbated during the COVID-19 pandemic.

An evaluation of *headspace* identified relatively high unit costs, with an average cost of $339 per occasion of service in 2013–14, and significant variation across centres ranging from $136 to more than $1000 [6]. Such disparities, alongside evidence of budget surpluses, suggest possible inefficiencies in service delivery. While some variation may be explained by contextual factors, it was recommended that centre-level operational practices be reviewed to ensure more efficient use of limited resources.

A recent article by Hickie and colleagues (2025) critically examines the role and impact of *headspace*, highlighting two diverging narratives [7]. On one hand, *headspace national* reports that most young people who attend present with relatively low levels of clinical disorder, functional impairment, or risk, with many receiving only one to three sessions of brief support. On the other hand, independent academic analyses (from the Brain and Mind Centre and others) indicate that around 60–75% of young people actually have moderate to high levels of clinical and psychosocial complexity, requiring more substantive interventions.

Across both national and academic datasets, clinical outcomes appear limited: fewer than one-third of young people show significant improvements in functioning, while the majority remain unchanged or deteriorate. The paper argues that while *headspace* has improved access to care, its current focus on brief, low-intensity interventions is misaligned with the more complex needs of many *headspace* clients. The authors conclude that Australia’s youth mental health system requires reconfiguration towards more comprehensive, measurement-based, and personalised care models, capable of addressing early stages of major mental disorders and improving long-term outcomes.

Early identification and intervention are paramount to mitigating long-term adverse outcomes, yet many young people who are experiencing distress remain unidentified and unconnected to support services. Traditional mental health service delivery models tend to rely on self-referral or crisis presentation, which may miss a substantial ‘hidden cohort’ of at-risk individuals. The premise of the Albury Project Mental Health pilot initiative was whether this gap could be addressed through an innovative, proactive, flexible and community-embedded approach that can identify needs upstream and provide a more systemic but low-threshold level of support.

The purpose of this article is to present information on the implementation and initial outcomes of the Albury Project Mental Health Pilot, a six-month initiative undertaken between July and December 2024, designed to test the adaptability of the Community of Schools and Services (COSS) Model to proactively identify and support youth experiencing psychological distress but who are not connected with mental health services or support. This is a single-site, single-arm exploratory pilot with no control group. The funding available from the New South Wales Department of Communities and Justice was limited, and the time frame was only six months. However, all parties considered this pilot initiative as a pioneering first step in the development of an extension of the COSS Model.

### 1.1. The Community of Schools and Services Model

The Community of Schools and Services (COSS) Model is an innovative early intervention service-delivery and reform-orientated model developed by *Upstream Australia.* By proactively identifying and supporting vulnerable young people and their families, the COSS Model reduces risk factors and increases protective factors, providing assistance where youth/family issues are heading towards a crisis, such as possible homelessness, early school leaving, and mental health problems.

The COSS Model is an exemplar of place-based collective impact, first articulated by Kania and Kramer (2011) as “the commitment of a group of important actors from different sectors to a common agenda for solving a specific social problem” [8]. While the goal of collaboration in the human services sector is not a new idea, collective impact initiatives are different from the status quo of targeted programs, by having a “centralized infrastructure, a dedicated staff, and a structured process that leads to a common agenda, shared measurement, continuous communication, and mutually reinforcing activities among all participants” (pp. 36–38). Collective impact is especially suited to addressing entrenched or ‘wicked’ problems that require systemic change across multiple levels and sectors as it shifts the focus from isolated interventions to coordinated systems change, with an emphasis on outcomes and sustained collaboration over time. An in-depth discussion of the COSS Model as place-based collective impact initiative is outside the scope of this article see [9,10,11,12] for more details.

#### COSS Model Core Features

The core features of the COSS Model are outlined following:

Community Collaboration and Shared Vision

At the heart of the COSS Model is genuine collaboration across the community. Schools, youth services, family support agencies, and other organisations work together in ways that move well beyond traditional referrals. Formal and informal partnerships enable joint planning, shared decision-making, and coordinated service delivery. This collaboration is guided by a common agenda: a shared understanding of homelessness as a systemic issue and a collective commitment to addressing it. While each organisation undertakes distinct activities, these efforts are aligned and mutually reinforced, ensuring that all contributions add up to more than the sum of their parts.

Early Identification and Proactive Screening

A key innovation of the model is its proactive approach to identifying young people at risk. Using a population health screening methodology, the COSS Model screens the entire secondary school population of a community each year. This is made possible through the proprietary Australian Index of Adolescent Development (AIAD) survey, which has been rigorously tested and refined over time. The AIAD Survey, developed by *Upstream Australia*, collects identifiable student data across demographics and several validated indicators including but not limited to the:

- *At-Risk of Homelessness Indicator*, a proprietary five-item Likert scale assessing risk across five dimensions—attitude, disposition, behaviour, relationships, and environment—externally validated [13].
- *Kessler Psychological Distress Scale (K-10)*, a widely used validated scale for identifying psychological distress that suggests the possibility of mental health issues [14]. The scale does not provide diagnostic information about specific mental health conditions.
- *Personal Wellbeing Index (PWI) contains* seven items of satisfaction, each one corresponding to a quality-of-life domain such as: standard of living, health, life achievement, personal relationships, personal safety, community-connectedness, and future security. The AIAD Survey uses the PWI-SC (but generically referred to in this article as the PWI), which is a simplified youth-friendly version of the PWI, using the term ‘happiness’ rather than ‘satisfaction’ [15].

AIAD survey data on identified at-risk students is combined with local insights and post-survey screening interviews to inform decision-making about intake into the COSS Model interventions. This methodology enables hidden populations of risk to be identified and interventions to be provided before crises occur, ensuring that support is available as early as possible.

Flexible Practice Framework and Early Intervention Support

The COSS Model builds a flexible framework of supports around young people and their families. The young person remains the primary client, but the model recognises that lasting solutions often require working directly with families as well. Interventions are tailored through a tiered system of support: from light-touch monitoring and secondary consultations (Tier One) to more intensive case management (Tier Two), through to fully wrap-around, multi-service support (Tier Three). This flexibility ensures that responses are both proportionate to the level of need and integrated across domains such as education, housing, and mental health.

The COSS Model Practice Framework was developed to operationalise ‘need’ with more nuance and flexibility than is commonly the case in the social services sector or the homelessness services system. It is a form of ‘triage’, a practice well understood in the health field. The COSS Model Practice Framework provides for a three-level response operationalized in a way that makes it possible for a ‘triage-like’ flexibility so that young people in need are prioritised according to need and receive timely interventions appropriate to the nature and level of their need for support.

Robust Use of Data, Monitoring and Outcomes

Data is not an afterthought but an embedded feature of the COSS Model. Longitudinal monitoring tracks individual progress and community-level outcomes, allowing both practice and system design to be continually refined. Shared measurement tools keep participating organisations aligned and accountable, while continuous feedback loops ensure that lessons from data are fed directly back into service delivery. The result is a practice environment that is evidence-informed, adaptive, and capable of demonstrating impact—whether in, for example, reduced risk, improved educational engagement, or lower reliance on crisis services.

Backbone Support, Leadership and Fidelity

Successful implementation of the COSS Model requires dedicated backbone support. Each site is anchored by a local coordinator and leadership team, providing the governance and operational infrastructure for the work. In addition, *Upstream Australia*—the innovation developer—is a platform that provides fidelity guidance to ensure amid community-level adaptation that the core features are not compromised, as well as in-built data management systems to support the measurement and evaluation of outcomes. This backbone function is important: complex collective impact initiatives require sustained leadership, coordination, and technical expertise if they are to deliver consistent results across multiple sites [16].

### 1.2. From Program Thinking to System Reform

The COSS Model represents a deliberate shift away from traditional program-based thinking. Rather than adding another service into an already fragmented landscape, it requires local communities—schools, services, local government, and young people themselves—to align around a shared vision and a collective structure for prevention. This is not about stand-alone initiatives, but about reconfiguring how the system itself operates.

The COSS Model is about community-level system reform. It establishes a support and service delivery platform for prevention and early intervention that is coordinated, data-driven, and locally led—shifting the locus from crisis management to prevention. By weaving schools, families, and community services into an integrated system of support, the COSS Model demonstrates that communities can move upstream, preventing homelessness or other adverse issues, thus delivering measurably significant, long-term outcomes for vulnerable young people.

### 1.3. Moving Beyond Referral-Only-Based Approaches

Many early intervention programs for young people continue to rely on referrals from teachers, parents, or the young people themselves. While these pathways can connect some at-risk youth to support, research consistently shows that they are insufficient on their own. A systematic review of school-based programs for the identification of children and young people with mental health difficulties concluded that “universal screening may be the most effective method of identification” [17]. Compared to less formal processes such as teacher or parent referrals, or self-referrals, systematic school-based approaches identify a greater proportion of children and young people with emerging difficulties [17,18,19,20,21,22,23].

#### 1.3.1. The Limits of Teacher and School Staff Referrals

It is sometimes suggested that teacher identification could serve as a substitute for systematic screening. However, the evidence indicates otherwise. Teachers often report that they are not equipped to implement systemic approaches to identification and frequently under-identify early symptoms of mental health disorders [24,25,26]. One study comparing teacher referrals to a structured teacher-rated screening tool found that only about half of the students at risk of emotional and behavioural difficulties were correctly identified [17]. While combining universal screening with teacher input can improve accuracy, referral-only models consistently leave many vulnerable young people unidentified.

#### 1.3.2. The Problem with Relying on Only Self-Referrals and Direct Referrals

Relying on young people to self-identify, or on trusted adults to notice and refer them, introduces further limitations. Many adolescents are adept at concealing or minimising problems, especially if they fear stigma or unwanted intervention. Others do not disclose difficulties until situations have escalated, by which point opportunities for early intervention are diminished. Referral processes can also be shaped by adult biases—whether conscious or unconscious—where prior perceptions of a young person influence judgements about risk. While some young people who self-refer to high-quality programs may benefit, referral-based systems cannot claim to systematically identify risk across an entire school or community. Without a population-level approach, large numbers of at-risk young people remain invisible to services.

### 1.4. Why Universal Screening Matters: The COSS Model Approach

Universal screening addresses these limitations directly. Though it involves additional costs, specialist data analysis, and reporting, screening provides a structured, evidence-based methodology for systematically identifying risk across a whole population. When combined with local knowledge and engagement interviews, universal screening becomes an even more powerful tool for early intervention, enabling hidden populations of at-risk young people to be identified before problems escalate.

For this reason, the COSS Model does not rely exclusively on referrals. Instead, it uses a population screening methodology. This enables communities to identify risk at scale, identify young people at risk, including hidden populations of risk, link young people to support earlier, and build a proactive system of intervention. By embedding universal screening at the centre of its methodology, the COSS Model overcomes the flaws of referral-only approaches and provides a more reliable foundation for prevention and early intervention.

### 1.5. The Albury Project

The Albury Project, located in Albury, New South Wales (NSW), is an established COSS Model community with a current funded focus on youth homelessness prevention and early intervention. Albury is a regional city on the northern bank of the Murray River, across from the Victorian city of Wodonga on the southern bank, that together form the twin-city region of Albury–Wodonga straddling the NSW–Victorian border, positioned some 554 kilometres from Sydney, the capital of NSW, and 326 kilometres from Melbourne, the capital of Victoria. In many ways, Albury–Wodonga is a twin-city with several shared services and amenities; however, many social and community services and programs are directly controlled by and accountable to departments located in either state jurisdiction, with their own policy directions and targets.

Albury City has an estimated resident population of 58,317 (as of June 2024), while the broader Albury–Wodonga twin-city region has a combined population of just over 100,000 [27,28]. Compared with metropolitan areas, the region has a lower proportion of high-income households and a higher proportion of households in lower income brackets, reflecting pockets of socio-economic disadvantage [29]. These demographic and structural factors form an important backdrop for the implementation of the Albury Project as a place-based collective impact initiative.

The Albury Project has been led by a strong community collective comprising the local COSS lead agency, *Yes Unlimited*, the three participating Albury public secondary schools, together with other partners, including Albury City Council, *headspace* (youth mental health service), and Upstream Australia, the developer of the COSS Model. Additionally, the project has benefited from strong support from senior officers in the local area offices of both the Department of Communities and Justice and the Department of Education. Population screening, a core foundation of the COSS Model, has been successfully implemented annually since 2019.

The Albury Project has demonstrated significant reductions in the risk of homelessness and entry into the local homelessness service system. Findings reveal that: (a) when COSS Model support is delivered to identified at-risk students, 40–50% of individuals are no longer at such high risk of homelessness 12 months later; (b) only 3–5% of students identified as at risk of homelessness and supported through the COSS Model sought assistance from local homelessness services in the following two years; and (c) the flow of adolescents (12–18 years) into the local homelessness services was reduced by 40% from 2019 to 2023 [15].

### 1.6. The Albury Project Mental Health Pilot

While best known for early intervention and prevention of youth homelessness, the COSS Model’s robust architecture and methodology—encompassing population-level screening, a flexible practice framework, and embedded outcome measurement—is potentially a multi-issue model that can be extended to other domains of youth disadvantage, including youth mental health.

The Albury Project Mental Health Pilot adapted the existing COSS Model’s population screening and support methodology to address youth mental health issues. The pilot specifically aimed to:

- Scope the current need for and demand for mental health services among young people within the project’s reach.
- Provide low-level, brief interventions for identified young people.
- Test the integration of a mental health response within the existing Albury Project framework.

This pilot initiative was designed to explore the feasibility and impact of a proactive, data-informed approach to supporting young people with emerging mental health concerns who were otherwise disengaged from formal services by leveraging the existing COSS Model architecture and methodology.

## 2. Materials and Methods

This section describes how the Albury Project Mental Health Pilot was designed and implemented, including the adaptations made to the COSS Model, the processes used to identify and engage participants, and the methods applied to collect and measure outcomes.

### 2.1. Procedure: Adapting the COSS Model for the Mental Health Pilot

Adapting the COSS Model for this pilot involved some modification from the usual COSS Model practice, particularly around the population screening methodology, the COSS practice framework and tier ratings as discussed following.

#### 2.1.1. COSS Model Population Screening and Support Methodology for the Mental Health Pilot

**Figure 1:**
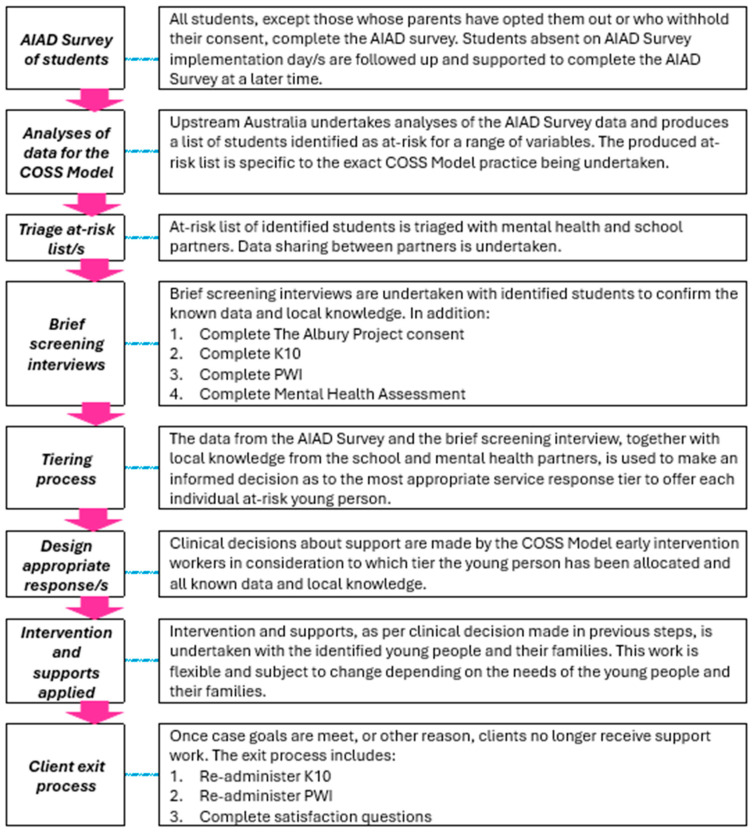
COSS Population Screening and Support Methodology for the Albury Project Mental Health Pilot.

The COSS Model population screening and support methodology employed for a youth homelessness focus was adapted for the Mental Health Pilot, see Figure 1. A post AIAD Survey interview tool was specifically designed for the Mental Health pilot. This interview was not a research-type interview but an interview to confirm the AIAD Survey results and to ascertain qualitative details of the young person’s current situation with a view to a future offer of support. This interview tool was adapted from the existing The Albury Project interview tool. The interview included a mental health risk assessment for all students and safety plan options.

#### 2.1.2. The COSS Practice Framework and Tier Ratings for the Mental Health Pilot

**Figure 2:**
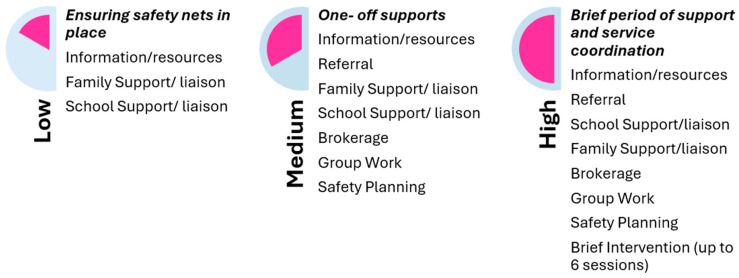
COSS Practice Framework and Tier Ratings for the Albury Project Mental Health Pilot.

The COSS Model Practice Framework and Tiers Ratings have been adapted for the Mental Health Pilot as demonstrated in Figure 2.

Given the short timeframe and limited resourcing, the pilot adopted a modified tiering approach that differed from the established practice of The Albury Project. In the pilot, young people requiring the highest level of intervention received a brief period of support. This contrasts with The Albury Project, where young people assessed at the highest level typically receive longer-term, wraparound case management involving multiple services. The brevity of the pilot interventions necessarily shaped both the scope and the depth of support provided.

Despite these constraints, interventions were tailored to tier allocations and addressed immediate needs. Safety planning was the most common form of support, provided to the majority of participants. Many young people were also linked with existing networks and supports, including family connections and school counselling services. A smaller number were referred to specialist mental health services such as *headspace*, while some accessed brokerage funding to address practical barriers. Others were provided with targeted information and resources to support self-help and facilitate future service access.

The pilot also trialed group-based support. Six participants engaged in a targeted, brief group intervention program built around the Orygen Brief Intervention Youth Mental Health Toolkit. This program covered practical wellbeing topics—including sleep hygiene, communication, and healthy relationships—while incorporating interactive activities such as cooking. These activities combined skill development with relational and experiential learning, aligning with the brief intervention focus of the pilot.

### 2.2. Pilot Participants

This section outlines how young people were identified and screened for inclusion in the Albury Project Mental Health Pilot and provides a summary of the characteristics and presenting issues of the pilot cohort.

#### 2.2.1. Determining Pilot Eligibility, Screening, and Tiering

The Albury Project Mental Health Pilot focused on young people who completed the AIAD Survey in Term 1, 2024 and who were not flagged at-risk of homelessness but had K10 indicator scores in the risk range (30–50) and had not received mental health support in the previous three months. The COSS Model operationalises two K-10 risk categories: scores of 30–39 as the risk category, and scores of 40–50 as the high risk category. Due to limited resources and to ensure a viable cohort size, K-10 scores of 39–50 were used in the pilot. To ensure a viable cohort within available resources, the pilot adopted a threshold of K10 ≥ 39.

Eligible students were cross-referenced with existing client lists for The Albury Project and *headspace*. Young people currently engaged in either service (*n* = 8) were excluded from the pilot, resulting in 45 students progressing to interview-based screening and tiering. Through this process, six students were assessed as “No Response” due to already having adequate mental health support in place (e.g., private psychology). Thirty-nine students received support through the pilot, allocated to the following tiers: low (17), medium (12), and high (10). Of the 39 pilot clients, 8 had an initial K10 score of 39 and 45 had scores of 40 or above.

The selection threshold and the exclusion of students already engaged with services likely resulted in a cohort with relatively higher symptom severity and may limit generalisability with students with lower-level symptoms or those currently receiving care. The exclusion of students already engaged with services (e.g., *headspace* or private psychology) effectively constituted a “treatment as usual” group who were outside the scope of the pilot, as the initiative specifically aimed to engage young people with unmet mental health needs who were not currently receiving support.

#### 2.2.2. The Pilot Cohort Characteristics

The pilot program clients comprised 39 students across three public secondary schools in Albury, NSW. The majority of pilot clients identified as female (*n* = 26), with a lower number of male clients (*n* = 9), and 1 person identifying as non-binary. Two clients in the pilot preferred not to share their gender identity.

Most clients were in Year 7 (*n* = 9), Year 8 (*n* = 9), and Year 9 (*n* = 10) in 2024, with fewer in Year 10 (*n* = 5), Year 11 (*n* = 4), and Year 12 (*n* = 2). On 1 July 2024, participants were aged between 12 and 17 years, with the largest groups aged 14 years (*n* = 9), 13 years (*n* = 8), and 12 years (*n* = 7), followed by those aged 16 years (*n* = 6), 15 years (*n* = 5), and 17 years (*n* = 4). The mean average age of the cohort was 14.1 years.

Nearly a quarter (*n* = 9; 23.07%) of clients identified as Aboriginal or Torres Strait Islanders. The majority of clients were born in an English-speaking country. In total, three students reported they were born in a non-English-speaking country, of whom one had been in Australia for less than five years.

**Figure 3:**
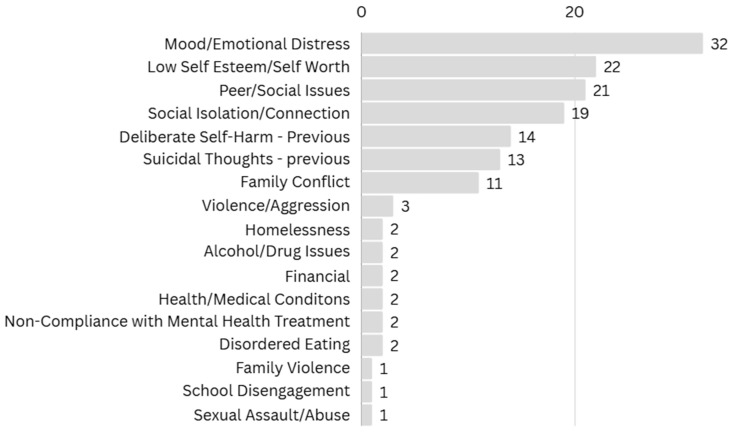
Self-Reported Presenting Issues at Pilot Intake.

At the point of intake screening into the pilot, common presenting issues included mood/emotional distress (*n* = 32) and low self-esteem/self-worth (*n* = 22), see Figure 3. All young people had multiple presenting issues. Peer/social issues and social isolation/connection were also frequently reported. Notably, many young people disclosed previous suicidal thoughts (*n* = 13) and/or previous self-harm (*n* = 14). Although the cohort was not at-risk of homelessness at the time of the 2024 AIAD Survey implementation (February 2024), two young people reported homelessness as a presenting issue during the pilot screening interview (July 2024), highlighting the dynamic nature of vulnerability.

### 2.3. Data Collection Procedure and Outcome Measurement

As discussed above, participants in the Albury Project Mental Health Pilot were identified through the 2024 administration of the AIAD Survey as part of regular practice in The Albury Project. Eligibility criteria were based on scores from the Kessler Psychological Distress Scale (K10) cross-referenced with AIAD Survey items relating to the presence or absence of mental health support within the preceding three months.

Pilot outcomes were assessed at two distinct time points. Short-term outcomes were collected by the mental health early intervention worker at the point of client exit, within one to five months of engagement (July–December 2024). December 2024 marked the end of the intervention period of the pilot program. Measures collected at this time included re-administration of the Kessler Psychological Distress Scale (K10) and the Personal Wellbeing Index (PWI), both of which are used routinely in the AIAD Survey, alongside structured questions about their engagement with education and their homelessness risk status.

Medium-term outcomes were assessed through the subsequent cycle of the AIAD Survey administered in Term 1, 2025. This provided a longer-term comparison of individual participants’ scores on key indicators, including the Kessler Psychological Distress Scale (K10), Personal Wellbeing Index (PWI), and the At-Risk of Homelessness Indicator. Comparing pilot participants’ 2024 AIAD Survey responses with their 2025 AIAD Survey responses allowed for an examination of change over a 12-month period. However, at the end of 2024, four students graduated secondary school or successfully transferred to TAFE and were therefore not able to undertake the AIAD Survey in 2025. Another nine students declined to undertake the AIAD Survey in 2024, although they remained enrolled at school. This left twenty-six pilot clients who undertook the 2025 AIAD Survey. A small number of students completed the survey but not all the items on the key indicator scales and so were cases of missing values.

Taken together, this approach to measurement allowed for both short-term program-level outcomes (immediate changes in psychological distress, personal wellbeing, homelessness risk status, and engagement with education) and medium-term indicators of impact (changes in psychological distress levels, personal wellbeing, and homelessness risk status across a 12-month timeframe).

As the pilot cohort was small (*n* = 39), the application of inferential statistical analysis was not appropriate. This is acknowledged as a limitation, as it restricts the extent to which findings can be generalised beyond the pilot sample. Small sample sizes reduce statistical power, increase the likelihood of measurement error; as such, only moderate to large effects are likely to be evident. In addition, the influence of outliers is proportionally greater in smaller cohorts, making it important to carefully examine difference scores to ensure that individual cases do not disproportionately affect observed trends. Nevertheless, such constraints are common in exploratory studies such as this pilot project, where the main aim is to generate preliminary insights and assess feasibility from practical experience in order to inform the design of a larger-scale program of intervention and possible random control trial evaluation methodology.

Another limitation of this pilot study is the lack of systematic qualitative data gathered from the pilot participants and their parents/family and school wellbeing staff. The main reason was that with limited funding, the pilot prioritised the practice work undertaken by the mental health early intervention worker. A more systematic and substantial body of client qualitative data would be useful for a more in-depth record of client’s lived experience of the pilot program.

### 2.4. Ethical Conduct and Consent

All data was collected and managed under the ethical standards of the National Statement on Ethical Conduct in Human Research, overseen by *Upstream Australia*, as part of the usual COSS Model practice in the Albury Project.

The AIAD survey is used as a key part of annual screening for risk in COSS Model communities. There are a range of consents, from the initial opt-out option for parents/guardians, as to whether they object to their children undertaking the survey, to active consent by students, as to whether they want to take the survey, and further consent from the identified young people, for a post-survey follow-up interview and for the offer of support. Parents/guardians receive written information about the local COSS Model implementation prior to the implementation of the AIAD Survey in their child/ren’s school. Parents/guardians are able to raise any potential questions about the local COSS Model implementation and/or AIAD Survey with Upstream Australia, the local COSS community services agency prior to AIAD Survey implementation. Young people are reminded on AIAD Survey implementation day in their school that participation is voluntary. Given that the purpose of the process and the COSS Model is to identify risk before crisis and ameliorate cognate risks, ethics approvals have been granted for Upstream Australia to collect and manage identifiable data for the COSS Model sites. Care and strict operational practices are followed to safeguard privacy, while noting that efficient and effective early intervention depends on being able to identify vulnerable individuals to prevent crises from occurring.

## 3. Results

This section reports the outcomes of the pilot across five domains: (1) participant engagement and program exits, (2) engagement with education, (3) risk of homelessness, (4) psychological distress as measured by the Kessler Psychological Distress Scale (K10), and (5) subjective wellbeing as assessed using the Personal Wellbeing Index (PWI). Results are presented for the total cohort and by tier level (Tier 1: lower intensity needs; Tier 2: moderate needs; Tier 3: higher needs). Outcomes are examined at three time points—baseline/pre-intervention (February 2024), short-term follow-up (August–December 2024), and medium-term follow-up (February 2025), see Table 1. For each measure, paired data are used where available to assess changes over time, and proportions meeting risk thresholds are reported, see Table 2. The findings provide insight into both immediate and potentially sustainable impacts of the intervention on homelessness risk, psychological distress, and overall wellbeing.

### 3.1. Participant Engagement and Pilot Exits

Across the duration of the pilot, young people demonstrated high levels of engagement and retention. All participants exited from the pilot prior to the end of 2024, and 87% (*n* = 34) completed a planned and purposeful exit, occurring after their case goals had been achieved and when the mental health worker assessed them as stable within their school and family environments. Only a small number exited earlier than anticipated, and no unplanned disengagements occurred due to refusal of support.

### 3.2. Engagement with Education

At the final 2024 check-in, all pilot clients remained enrolled in secondary school (*n* = 35) during 2024. At the end of 2024, one student has successfully transition to another education option (TAFE) and three had graduated (*n* = 4). The mental health worker noted that at the end of year check-in, 10 clients had attended secondary school less than 70% over the previous month.

### 3.3. Risk of Homelessness

At baseline (February 2024), none of the 36 young people who completed the At Risk of Homelessness Indicator in the AIAD Survey were in the risk range (score 7–10). Average mean scores were low across tiers:

- Total cohort: 3.0
- Tier 1: 2.94
- Tier 2: 2.88
- Tier 3: 3.2

By the short-term follow-up (August–December 2024), a small number of young people were assessed by workers as being in the risk range (3 of 37; 8.1%). Two were in Tier 1 (12.5%), and one was in Tier 3 (11.1%). No Tier 2 participants were identified as at-risk at this time. Because worker assessments were based on engagement with the pilot clients, no mean averages are available as the At Risk of Homelessness Indicator was not utilized and are, therefore, not reported at this wave.

By the medium-term follow-up (February 2025), homelessness risk remained very low across all cohorts. Only one young person in Tier 1 and one young person in Tier 3 fell within the risk range. Across the full sample, only one participant (4%) met the risk threshold. Average mean risk scores decreased from baseline in Tier 1 (2.94 → 1.54) and Tier 2 (2.88 → 1.50) and increased slightly in Tier 3 (3.3 → 3.5).

Overall, homelessness risk remained low across all groups over time. Among Tiers 1 and 2, risk scores improved (i.e., decreased). Tier 3 showed greater variability, with two young people registering elevated risk at different time points, consistent with their higher baseline needs profile.

### 3.4. Psychological Distress

Psychological distress, as measured by the Kessler Psychological Distress Scale (K10) was extremely high at intake. All 39 young people (100%) recorded K10 scores in the risk range (30–50). Average mean scores were high and consistent across tiers:

- Total cohort: 42.3
- Tier 1: 41.76
- Tier 2: 42.58
- Tier 3: 42.9

A total of 33 young people had paired pre/post K10 data for the short-term period. Of these, 30 young people (90.9%) showed reduced distress relative to baseline. All Tier 1 and Tier 2 participants with paired data experienced improvement (100%). In Tier 3, 7 of 10 young people improved (70%).

Average mean K10 scores decreased substantially:

- Total cohort: from 42.3 → 27.3
- Tier 1: 41.76 → 22.3
- Tier 2: 42.58 → 27.6
- Tier 3: 42.9 → 33.5

Despite large mean reductions, fourteen participants (42.4%) of the overall group remained in the clinical range.

Twenty-three young people had paired pre/post K10 data at medium-term follow-up. Of these, 86.9% showed sustained improvement since baseline:

- Tier 1: 100% improved
- Tier 2: 100% improved
- Tier 3: 50% improved

Average mean medium-term K10 scores were:

- Total cohort: 29.52
- Tier 1: 25.3
- Tier 2: 24.57
- Tier 3: 42.33

Tier 3 showed a return toward baseline levels for several participants, indicating ongoing elevated distress despite earlier short-term gains.

### 3.5. Subjective Wellbeing

Subjective wellbeing was measured using the Personal Wellbeing Index (PWI). Overall wellbeing at baseline was low but not in the low or very low (i.e., the risk) range (total average mean = 45.64). Forty percent (15 of 37) scored below the wellbeing risk threshold (<39). Tier 3 presented the lowest average mean wellbeing score (38.2); however, half of Tier 3 participants (5 of 10) were in the risk range.

Among the 31 young people with paired pre/short-term data, 77.4% reported improved wellbeing. Improvement by tier is as follows:

- Tier 1: 100% improved
- Tier 2: 66.7% improved
- Tier 3: 60% improved

Average mean wellbeing scores increased across tiers:

- Total cohort: 45.64 → 55.69
- Tier 1: 44.5 → 63.53
- Tier 2: 54.09 → 57.8
- Tier 3: 38.2 → 43.4

Twenty-four young people had paired pre/medium-term wellbeing data; 70.8% demonstrated improved scores relative to baseline. By tier:

- Tier 1: 70% improved
- Tier 2: 75% improved
- Tier 3: 66.7% improved

Average mean scores remained above baseline for all tiers:

- Total cohort: 45.64 → 55.8
- Tier 1: 44.5 → 57.45
- Tier 2: 54.09 → 61.37
- Tier 3: 38.2 → 45.33

## 4. Discussion

The Albury Project Mental Health Pilot provides early evidence that the Community of Schools and Services (COSS) Model can be adapted to proactively identify and support young people experiencing psychological distress who were not engaged in formal mental health services. In the context of a fragmented national youth mental health system, as discussed in the introduction, characterized by long wait times, brief interventions that often misalign with client complexity, and high levels of unmet need, this pilot project demonstrated the practicality of embedding mental health identification and responses within the collective impact framework of an existing COSS Model community site. Despite operating over a short timeframe, the pilot achieved its aims of scoping need, delivering brief tailored interventions, and integrating mental health responses within an established COSS infrastructure, yielding promising measurable improvements in psychological distress and strong engagement outcomes.

### 4.1. Identifying a Hidden Cohort Through Population-Level Screening

A key finding of the pilot was the identification of a “hidden cohort” of young people experiencing significant psychological distress who were not connected to mental health services. This group became visible only through the COSS Model’s population-level screening, implemented annually via the AIAD Survey. This is consistent with research showing that universal school-based screening identifies substantially more young people with emerging difficulties than referral-only or self-referral pathways, and that teacher or parent identification alone often underestimates early symptoms, e.g., [17,18,19,20,21,22,23,24,25,26].

By embedding screening within a mature COSS environment, with strong school partnerships, shared measurement systems, practitioner-led engagement interviews, and established relationships with headspace and youth services, the pilot achieved efficient triage and reduced barriers commonly encountered when establishing new community initiatives. The ability to detect unmet need at scale reinforces the value of population-level methods as part of an upstream mental health response.

### 4.2. Psychological Distress: Meaningful Short- and Medium-Term Improvements

Psychological distress decreased substantially across the cohort. The proportion of participants in the K10 risk range reduced from 100% at intake to 42% at the short-term follow-up and remained substantially improved at 12 months. These changes are clinically meaningful and consistent with evidence that early intervention can mitigate escalation of mental health problems in adolescence [30,31]. Tier-level patterns further support the value of a differentiated response. Participants with lower or moderate levels of need, allocated to Tiers 1 and 2, showed the largest and most stable improvements. Tier 3 participants demonstrated early gains but more variability over time, reflecting evidence that young people with cumulative adversity or complex psychosocial needs typically require longer-term or more intensive intervention [32]. These findings illustrate that while a brief, flexible intervention is often sufficient for many young people, others with greater or more complex needs require sustained or escalated support.

### 4.3. Low-Intensity, Proportionate Support Within a Tiered Practice Framework

A notable insight from the pilot is that high psychological distress does not necessarily equate to a need for specialist clinical intervention. Many young people benefited from low-intensity, relational, and proactive support focused on foundational issues such as peer conflict, isolation, relational stress, and emerging anxiety. These findings echo critiques that current national youth mental health models sometimes provide brief, low-intensity support to young people with higher needs while simultaneously over-medicalising lower-need presentations [7].

The COSS Model’s flexible tiered response allowed supports to be proportionate to need, ranging from light-touch monitoring to more intensive case management, with agile responses tailored to individual circumstances. High engagement and retention rates (87% planned exits) indicate strong acceptability of this approach within a familiar and well-established COSS Model context. Also, the cohort differed from the traditional Albury Project youth homelessness cohort, showing higher school engagement and lower levels of family dysfunction, highlighting the importance of screening in revealing subclinical but significant risk profiles that might otherwise remain undetected.

### 4.4. Stability in Homelessness Risk and Multi-Issue Responsiveness

Homelessness risk remained low throughout the study period, with only small fluctuations, at later follow-up. This stability likely reflects a combination of protective family or school factors, early support, and the model’s multi-issue orientation. Importantly, integrating mental health intervention did not detract from the COSS Model’s original purpose (i.e., youth homelessness prevention and early intervention) but rather expanded its capacity to address overlapping domains of need. These findings strengthen the case for the COSS Model as a multi-issue platform capable of addressing complex youth disadvantages beyond homelessness risk alone.

### 4.5. Implementation Strengths

The successful implementation of the pilot was strongly supported by the maturity of the existing COSS infrastructure. Well-established partnerships, routine data sharing, structured governance, and embedded triage processes enabled rapid and high-fidelity integration of mental health functions. This aligns with collective impact literature that emphasises the critical importance of backbone support, shared measurement, and collaborative structures in achieving significant sustainable outcomes, all features of a collective impact approach that must be an embedded and ongoing part of the work [33,34]. When these foundations are in place, scaling into additional areas of youth need, in this case mental health, can be achieved efficiently and with effectiveness.

### 4.6. Limitations

Several limitations warrant consideration. The pilot involved a small sample size, operated without a comparison group, and was conducted at a single site across three schools, limiting generalizability. The six-month implementation period, determined by funding constraints, restricted the ability to assess longer-term outcomes or sustainability. Some missing follow-up data, particularly within tier subgroups, reduces precision in reporting. Practitioner-reported short-term homelessness risk may involve subjectivity, though triangulation with standardised instruments at baseline and 12 months provides some mitigation. Differences in data collection settings (annual AIAD Survey administered in classroom settings versus one-to-one check-ins) may also have influenced responses. The discontinuation of pilot funding prevented further follow-up beyond the 12-month data point.

### 4.7. Implications and Future Directions

Despite these limitations, the pilot has provided some important insights for early intervention and youth mental health policy. It has demonstrated that population-level screening embedded within a COSS Model collective impact, place-based framework can detect unmet need that traditional referral pathways miss. It also shows that flexible, proportionate, school-community-based support can produce meaningful improvements in psychological distress, stabilize multi-issue risk, and reduce the demand pressure on overstretched clinical services such as *headspace*.

These findings support the potential scalability of the COSS Model as an integrated, multi-issue early-intervention platform spanning mental health, homelessness prevention and early intervention, educational engagement, and broader youth wellbeing. Future research should include multi-site implementation, more rigorous evaluation designs, and longer-term follow-up to determine sustainability, cost-effectiveness, and population-level impact.

## 5. Conclusions

In conclusion, the Albury Project Mental Health Pilot, although a small-scale limited exploratory project, has provided promising evidence for the potential of a proactive, population-level, place-based collective impact early intervention model for youth mental health complementary to the existing infrastructure. However, the Albury Project context for the pilot initiative underscores the importance of leveraging the COSS Model, the existing community-based collective impact framework already in operation, to systematically identify and support that ‘hidden cohort’ of young people at risk. Finally, a lesson for future funding from government departments is that future development of this approach will need sustained, cross-departmental funding. This is system change on the ground, away from the status quo of siloed, targeted programs from multiple government departments to a place-based collective impact approach as exemplified by Albury Project Mental Health pilot program initiative.

## Figures and Tables

**Table 1:** Risk Ranges and Change Percentages, across Tier Levels and Outcome Variables, Pre and Post Intervention.

Risk Ranges and Change Percentages, Across Tier Levels and Outcome Variables, Pre and Post Intervention	Total Cohort (*n* = 39 *)			Tier 1 (*n* = 17 *)			Tier 2 (*n* = 12)			Tier 3 (*n* = 10)		
Intervention Timing	Pre	Post	Post	Pre	Post	Post	Pre	Post	Post	Pre	Post	Post
Data Collection Timing	February 2024	August–December 2024	February 2025	February 2024	August–December 2024	February 2025	February 2024	August–December 2024	February 2025	February 2024	August–December 2024	February 2025
At Risk of Homelessness Indicator Indicator Score Range: 0–10 Indicator Risk Range: 7–10												
Data Tool Administered	AIAD	Worker Ax	AIAD	AIAD	Worker Ax	AIAD	AIAD	Worker Ax	AIAD	AIAD	Worker Ax	AIAD
Total Response (*n*=)	36	37	25	17	16	11	9	11	8	10	9	6
Mean average indicator score (total responses)	3	n/a	2	2.94	n/a	1.54	2.88	n/a	1.5	3.2	n/a	3.5
Total clients in risk range (*n*=)	0	3	1	0	2	0	0	0	0	0	1	1
Total clients in risk range (% of responses)	0	8.10	4.00	0	12.5	0	0	0	0	0	11.11	16.66
Mean average indicator score (total clients in risk range)	0	n/a	8.00	0	n/a	0	0	n/a	0	0	n/a	8.00
Total clients not in risk range (*n*=)	36	34	24	17	14	11	9	11	8	10	8	5
Total clients not in risk range (% of responses)	100.00	91.89	96.00	100.00	87.5	100.00	100.00	100.00	100.00	100.00	88.88	83.33
Mean average indicator score (total clients not in risk range)	3	n/a	1.75	2.94	n/a	1.54	2.88	n/a	1.5	3.2	n/a	2.6
Kessler Psychological Distress Scale (K10) Indicator Score Range: 0–50 Indicator Risk Range: 30–50												
Data Tool Administered	AIAD	Standalone K10	AIAD	AIAD	Standalone K10	AIAD	AIAD	Standalone K10	AIAD	AIAD	Standalone K10	AIAD
Total Response (*n*=)	39	33	23	17	13	10	12	10	7	10	10	6
Mean average indicator score (total responses)	42.3	27.3	29.52	41.76	22.3	25.3	42.58	27.6	24.57	42.9	33.5	42.33
Total clients in risk range (*n*=)	39	14	12	17	3	5	12	4	2	10	7	5
Total clients in risk range (% of responses)	100.00	42.42	52.17	100.00	23.07	50.00	100.00	40.00	28.57	100.00	70.00	83.33
Mean average indicator score (total clients in risk range)	42.3	37.07	38.66	41.76	22.3	34.3	42.58	36.5	33.5	42.9	39.28	45
Total clients not in risk range (*n*=)	0	19	11	0	10	5	0	6	5	0	3	1
Total clients not in risk range (% of responses)	0	57.57	47.82	0	76.92	50.00	0	60.00	71.42	0	30.00	16.66
Mean average indicator score (total clients not in risk range)	0	20.10	19.54	0	19.2	16.2	0	21.66	21.00	0	20.00	29.00
Personal Wellbeing Index (PWI) Indicator Score Range: 0–100 Indicator Risk Range: 0–39												
Data Tool Administered	AIAD	Standalone PWI	AIAD	AIAD	Standalone PWI	AIAD	AIAD	Standalone PWI	AIAD	AIAD	Standalone PWI	AIAD
Total Response (*n*=)	37	33	25	16	13	11	11	10	8	10	10	6
Mean average indicator score (total responses)	45.64	55.69	55.8	44.5	63.53	57.45	54.09	57.8	61.37	38.2	43.4	45.33
Total clients in risk range (*n*=)	15	7	4	8	2	2	2	2	0	5	3	2
Total clients in risk range (% of responses)	40.54	21.21	16.00	50.00	15.38	18.18			0	50.00	30.00	33.33
Mean average indicator score (total clients in risk range)	30.73	27.42	30.5	31.37	38.5	35.5	27.5	25.5	0	31	21.33	25.5
Total clients not in risk range (*n*=)	22	26	21	8	11	9	9	8	8	5	7	4
Total clients not in risk range (% of responses)	59.45	78.78	84.00	50.00	84.61	81.81				50.00	70.00	66.66
Mean average indicator score (total clients not in risk range)	55.81	63.3	60.61	57.62	68.09	62.33	60.00	65.87	100.00	45.4	54.85	55.25

* Cohort size for post-intervention (February 2025) reduced as 4 clients were no longer secondary school students in 2025 as they had graduated/moved to TAFE and were therefore ineligible to complete the AIAD Survey in 2025.

**Table 2:** Movement in Risk Scores as Measured by the K10 and PWI, across Tier levels, pre and post intervention.

Movement in Risk Scores as Measured by the K10 and PWI, Across Tier levels, Pre and Post Intervention		Total Cohort (*n* = 39 *)	Tier 1: Low (*n* = 17 *)	Tier 2: Medium (*n* = 12)	Tier 3: High (*n* = 10)
Kessler Psychological Distress Scale (K10)					
Pre-intervention: February 2024	Total Response (*n*=)	39	17	12	10
Post-intervention: August–December 2024	Clients with Pre & Post (August–December 2024) Responses (*n*=)	33	13	10	10
	Clients with improved/reduced risk in comparison to pre-intervention risk score (*n*=)	30	13	10	7
	Clients with improved/reduced risk in comparison to pre-intervention risk score (%=)	90.90	100.00	100.00	70.00
Post-intervention: February 2025	Clients with Pre & Post (February 2025) Responses (*n*=)	23	10	7	6
	Clients with improved/reduced risk in comparison to pre-intervention risk score (*n*=)	20	10	7	3
	Clients with improved/reduced risk in comparison to pre-intervention risk score (%)	86.95	100.00	100.00	50.00
Personal Wellbeing Index (PWI)					
Pre-intervention: February 2024	Total Response (*n*=)	37	16	11	10
Post-intervention: August–December 2024	Clients with Pre & Post (August–December 2024) Responses (*n*=)	31	12	9	10
	Clients with improved/reduced risk in comparison to pre-intervention risk score (*n*=)	24	12	6	6
	Clients with improved/reduced risk in comparison to pre-intervention risk score (%)	77.41	100.00	66.66	60.00
Post-intervention: February 2025	Clients with Pre & Post (February 2025) Responses (*n*=)	24	10	8	6
	Clients with improved/reduced risk in comparison to pre-intervention risk score (*n*=)	17	7	6	4
	Clients with improved/reduced risk in comparison to pre-intervention risk score (%)	70.83	70.00	75.00	66.66

* Cohort size for post-intervention (February 2025) reduced as 4 clients were no longer secondary school students in 2025 as they had graduated/moved to TAFE and were therefore ineligible to complete the AIAD Survey in 2025.

## Data Availability

The data underlying this study are client records from a community services organisation and contain sensitive personal information. Due to privacy, confidentiality, and ethical restrictions, the data cannot be made publicly available. Aggregated findings are reported within the article.

## Abbreviations

The following abbreviations are used in this manuscript:

ABS	Australian Bureau of Statistics
AIAD	Australian Index of Adolescent Development Survey
COSS	The Community of Schools and Services Model
K10	Kessler Psychological Distress Scale
NSW	New South Wales
PWI	Personal Wellbeing Index
